# The asymmetry of telomere replication contributes to replicative senescence heterogeneity

**DOI:** 10.1038/srep15326

**Published:** 2015-10-15

**Authors:** Thibault Bourgeron, Zhou Xu, Marie Doumic, Maria Teresa Teixeira

**Affiliations:** 1 INRIA Paris-Rocquencourt, Domaine de Voluceau, BP 105, 78153 Le Chesnay, France. UPMC University of Paris 6, JL Lions Lab., 4 place Jussieu, 75005 Paris, France; 2 Sorbonne Universités, UPMC Univ Paris 06, CNRS, UMR8226, Laboratoire de Biologie Moléculaire et Cellulaire des Eucaryotes, Institut de Biologie Physico-Chimique, 75005 Paris, France

## Abstract

In eukaryotes, the absence of telomerase results in telomere shortening, eventually leading to replicative senescence, an arrested state that prevents further cell divisions. While replicative senescence is mainly controlled by telomere length, the heterogeneity of its onset is not well understood. This study proposes a mathematical model based on the molecular mechanisms of telomere replication and shortening to decipher the causes of this heterogeneity. Using simulations fitted on experimental data obtained from individual lineages of senescent *Saccharomyces cerevisiae* cells, we decompose the sources of senescence heterogeneity into interclonal and intraclonal components, and show that the latter is based on the asymmetry of the telomere replication mechanism. We also evidence telomere rank-switching events with distinct frequencies in short-lived versus long-lived lineages, revealing that telomere shortening dynamics display important variations. Thus, the intrinsic heterogeneity of replicative senescence and its consequences find their roots in the asymmetric structure of telomeres.

Heterogeneity in cellular processes, whether programmed or stochastic (also called noise), has important physiological consequences in all organisms. In bacteria and microorganisms in general, phenotypic variability generally increases the fitness of the population both in normal and adverse conditions^1^,^2^,^3^,^4^. Developmental and, more generally, differentiation programs also aim at producing heterogeneity out of a uniform population of cells or even a single cell^5^,^6^,^7^,^8^,^9^,^10^.

In addition to the diversity of states reached by differentiation, many terminal differentiation processes also show important variations in time, from the first differentiation signal to the final cell fate commitment^5^,^11^,^12^,^13^. In unicellular organisms, this temporal heterogeneity may be viewed as a survival strategy by promoting flexibility in fluctuating environments. The fundamental role of noise in all these processes is further confirmed by the fact that noise can be controlled and evolved in genetic circuits to optimize its functionality^14^,^15^,^16^.

Replicative senescence is an asynchronous process well documented to display interclonal and intraclonal variations in different organisms^17^,^18^,^19^,^20^ and first described as a limit to the proliferation of human primary fibroblasts, the Hayflick limit^21^. An important step in understanding senescence onset and heterogeneity was to relate the Hayflick limit to the molecular mechanism of telomere shortening. Indeed, the ends of eukaryotic linear chromosomes, called telomeres, require a specialized mechanism for its maintenance, in most cases the holoenzyme telomerase. Without maintenance, as in primary fibroblasts or in telomerase-negative *Saccharomyces cerevisiae* cells, telomeres shorten and induce replicative senescence after many cell divisions. Many other factors affect and modulate replicative senescence and its inherent variability, including oxidative stress^22^,^23^,^24^, mitochondrial dysfunction^25^,^26^ and environmental stress^27^, which may act together and/or through telomeres^23^,^28^,^29^. Senescence may thus be seen as the outcome of complex interactions between metabolic and DNA-damage (including telomere dysfunction and attrition) pathways^30^, contributing to increasingly more variability over time or cell divisions^31^. This stochastic view of senescence stands in contrast with the idea that telomere shortening may represent a molecular clock mechanism for cell divisions. It is thus critical to assess the contribution of telomere-related variations to senescence heterogeneity.

In cells that express telomerase such as stem cells or most cancer cells in mammals, or unicellular eukaryotes, telomere length is a dynamic equilibrium between shortening and elongation by telomerase. Thus, in the presence of telomerase, the telomeres in a single cell encompass a broad range of lengths and an individual telomere, even in a clonal population of cells, displays an important length heterogeneity^32^. Many studies in different organisms converge to the idea that, when telomerase is absent, the average length of all telomeres is not critical for senescence onset. Rather, the shortest telomere(s) reaching a threshold may be the actual signal for senescence arrest^33^,^34^. Therefore, the length heterogeneity of the shortest telomeres, which may be greater than that of the others^20^, should strongly impact on senescence onset heterogeneity. At the molecular level, telomeres end with a 3′-end single-stranded DNA overhang^35^,^36^. Such a structure stems from the inability of the replication machinery to integrally replicate telomeres^37^,^38^. The direct consequence of this end-replication problem is the asymmetry of telomere replication, resulting in their overall shortening when no maintenance mechanism is present^39^. But whether this shortening mechanism by itself generates heterogeneity in telomere length has not been specifically addressed.

Here we aim at characterizing the different sources of senescence heterogeneity by a computational approach, using *Saccharomyces cerevisiae* as a model organism for telomere-induced senescence. In a recent study, we reported and quantified replicative senescence heterogeneity at the level of individual lineages, which was a first step to characterize heterogeneity by overcoming average measurements in population studies^40^. To analyze the heterogeneity observed in that study, we model the asymmetric replication of each telomere in a cell, using recent data on the molecular mechanism of the 3′-end overhang processing^41^, and the random segregation of the chromosomes in the two daughter cells. We consider senescence signal as the result of one or more telomeres reaching a length threshold and thus focus on telomere-dependent mechanisms of generating heterogeneity. By comparing simulations based on the mathematical model with the experimental data, we could decompose senescence heterogeneity into fundamentally distinct parts at different levels and evaluate their contribution.

We find that, in the model system, not only do cell-to-cell variations in the initial telomere length distribution contribute to senescence heterogeneity, but the molecular mechanism of asymmetric telomere replication also generates important intrinsic variations in telomere length dynamics. Therefore, a substantial part of replicative senescence heterogeneity may be structurally built in the telomere replication mechanism. As telomere structure and replication mechanism are broadly conserved in eukaryotes with some notable exceptions, this study provides a new molecular basis for phenotypic variations in multicellular organisms, with pleiotropic consequences in differentiation, aging and cancer emergence.

## Results

### A model of telomere replication and its impact on senescence onset

We define a lineage as the consecutive cells with a mother/daughter relationship, which we were able to track in a dedicated microfluidics-based live-microscopy assay^40^. The aim of the following model is to track the 32 telomeres of a haploid yeast cell at the single-cell level in the consecutive cells that make up a lineage, in the absence of telomerase. We note 
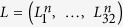
 the lengths of the 32 telomeres of a cell at the *n*^*th*^ generation in a lineage.

Telomere length dynamics is different in the presence and in the absence of telomerase. We first describe the steady state telomere length distribution obtained in the presence of telomerase, which we use as the initial distribution for all subsequent simulations (Fig. S1). We then describe a model based on the molecular mechanism of telomere replication and shortening in the absence of telomerase in individual lineages.

#### Initial steady state telomere distribution in the presence of telomerase

In the presence of telomerase, the telomeres in a cell evolve by two means: (i) they are shortened on average after replication, and (ii) they can be elongated by telomerase with a probability that decreases with increased length^42^. A model for the evolution of each length 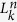, for *k* = 1, …, 32, was studied in^20^:
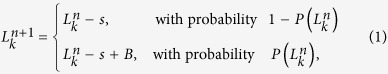
where *s* is the mean shortening rate at each division, *B* is the random elongation length due to the action of the telomerase and *P*(*L*) is the probability of action of telomerase as a function of telomere length. The variables *s, B, P*(*L*) do not depend on *k* or *n*. As in^20^, the parameter *s* is *s*et at *s* = 3.5 bp, *B* is a random variable following a geometrical law of parameter *p* = 0.026, and lastly the probability *P*(*L*) is fitted from the cumulative distribution function of empirical elongation lengths^42^, and expressed as follows:
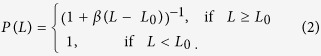


This probability of action of telomerase depends on two parameters *β* and *L*_0_ that can change from a yeast strain to another and a condition to another, depending on the *in vivo* activity of telomerase. We assume that the telomerase-positive initial cells we randomly pick for simulations have undergone a large number of divisions, thus reaching a steady state for their telomere lengths. Under this assumption, we can fit *β* and *L*_0_ from only two parameters of telomere length distribution: the mode and the skewness. The mode of telomere length distribution may be experimentally measured by telomere restriction fragment (TRF) Southern blot and the skewness is assumed to be the same as in^20^. For more details, see Materials and Methods. We are thus able to simulate steady state telomere lengths in a cell, corresponding to the experimental strain TetO2-*TLC1* yT528 used in^40^, which had a mode of 260 ± 16 bp. This steady state distribution is used in our simulations as the starting point distribution before telomerase inactivation (Fig. S1).

#### Telomere replication and shortening in the absence of telomerase

**Figure 1:**
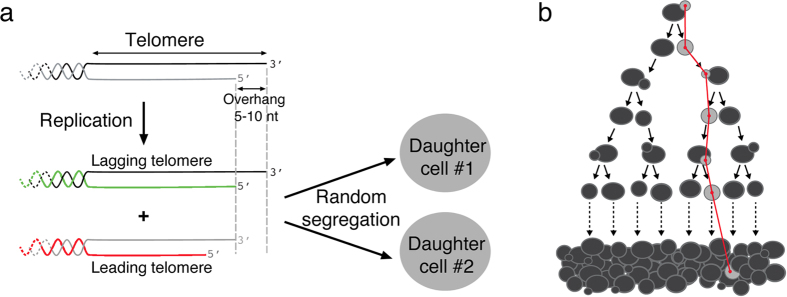
Model of telomere replication and segregation. (**a**) The molecular mechanism of telomere replication, at the basis of the mathematical model, is depicted. Replication of the parental telomere, harboring a 5–10-nucleotide 3′-end overhang, leads to two new telomeres of different lengths through the leading and lagging strand synthesis: one with parental length (black and green) and the other with shortened length (shorter by the overhang length; grey and red). Random segregation of chromosomes into the two daughter cells shuffles the set of 64 telomeres with each daughter inheriting 16 telomeres of parental length and 16 shortened ones. (**b**) Scheme of a branching model for cell division. Only one of two daughter cells at each generation is kept in the simulations as in the experiments, thus defining a lineage. Telomere length distribution is recorded for each cell in a lineage.

In the absence of telomerase, the average telomere length decreases at each division. At the level of individual telomeres, the mechanism of shortening stems from the DNA-end-replication problem^37^,^38^,^39^,^41^. In late S-phase, DNA replication gives rise to 64 telomeres: 32 lagging-strand telomeres of parental length and 32 leading-strand telomeres with shortened lengths^41^ (Fig. 1a). After random segregation of the two sets of chromosomes, each daughter cell receives 16 telomeres of parental length and 16 shortened telomeres, because one extremity of a chromosome is the result of the leading strand synthesis while the other is replicated by the lagging strand machinery. For the shortened telomeres, the loss of nucleotides corresponds to the length of the overhang of the original telomeres before replication, which is 5–10 nucleotides^41^ (Fig. 1a). We thus write the following mathematical model to describe telomere replication and segregation into daughter cells: for a random permutation *σ* of {1, …, 32}, for *k* = 1, …, 32,
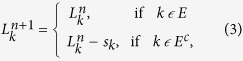
where 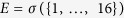, 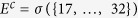 and 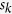 are uniform random variables on the set {5, 6, 7, 8, 9, 10}, corresponding to the overhang length. At generation n + 1, we keep track of only one of the two daughters, the succession of cell divisions thus defining a lineage (Fig. 1b). Another hypothesis was tested for the law of the random variables 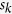, and will be explained below. For *n* ≥ 0, the smallest value among 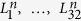 is denoted by *L1* and the second smallest value is denoted by *L2*. We model senescence onset as the consequence of a signal triggered by the weighted contribution of the two shortest telomeres reaching a threshold *Lmin*. Thus, the cell that stops dividing verifies the condition *L*1 + *a L* 2< *Lmin* for a constant value *a* in [0, 1]. The generation of senescence is then recorded. The dynamics of the shortening of the 32 telomeres in the successive cells of lineage is illustrated in the Supplementary Movie.

### The shortest telomere is the sole contributor to senescence onset signal

**Figure 2:**
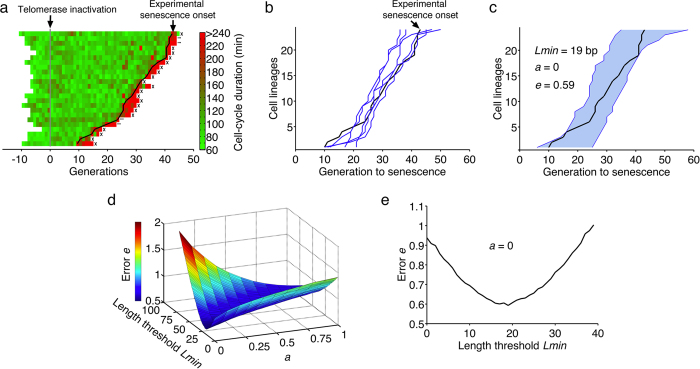
Fit of senescence onset timings. (**a**) The type A lineages from^40^ were used as experimental data. Display of *n* = 24 independent TetO2-*TLC1* cell lineages. Each horizontal line represents a lineage, and each segment is a cell cycle. Cell-cycle duration is indicated by the colour bar. The timings of transition into a senescent state were extracted (black line). (**b**) Representative set of 5 simulations of 24 senescence timings in independent lineages (blue lines, *Lmin* = 19 bp, *a* = 0) compared to the experimental senescence onset timings (black line), extracted from (**a**). (**c**) Envelope of *n* = 1000 simulations (blue-shaded area). (**d**) 3D-plot of the error of the fit *e* as a function of *a* and *Lmin*. (**e**) 2D-plot of the error of the fit *e* as a function of *Lmin* for *a* = 0.

As the model was built to analyze telomere length dynamics at the single-cell level, we used equally high-resolution data, namely senescence onset timing of individual yeast cell lineages, to validate it^40^. In that study, we clustered telomerase-negative lineages into two types depending on the presence of precocious and reversible cell cycle arrests before transition into senescence. The majority of the lineages, called type A, did not display these arrests and instead underwent normal cell divisions until a single sharp transition into senescence (Fig. 2a). Their progression into senescence was consistent with a model of progressive telomere shortening and sudden signal for senescence arrest. We thus extracted the experimental profile of senescent onset timings from the type A lineages and used these data to fit our model (Fig. 2a–c).

Evidence suggest that a single critically short telomere is sufficient to signal senescence^34^. In the model, the expression of senescence trigger as *L*1 + *a L*2 < *Lmin* was chosen to test this hypothesis by evaluating the contribution of the second shortest telomere to senescence signaling. We ran a total of >26 million simulations with different values for *a* and *Lmin* and found that the parameters that best fitted the experimental results were *a* = 0 and *Lmin* = 19 bp, with a root mean square deviation, which we simply call error of the fit, of *e* = 0.59 (Fig. 2d,e). An example of a fit with an error *e* = 1.7 (*a* = 0 and *Lmin* = 60 bp) is shown for reference in Fig. S2. This indicated that a contribution of the second shortest telomere to senescence signaling was unlikely and the shortest telomere reaching a length threshold was a sufficient condition to fit the data.

To test the robustness of the fit and assess whether the fit errors for *a* > 0 were significantly different from the minimal error found for *a* = 0, we changed the initial mode of telomere length distribution and tested 260 + 16 = 276 and 260 − 16 = 244 bp, corresponding to the upper and lower limits of the uncertainties of the initial telomere length (Fig. S3)^40^. Using these limit values, we found *a* = 0 for both conditions and *Lmin* = 35 and 2 bp, respectively (Fig. S3). Importantly, the errors in both cases (*e* = 0.60 and *e* = 0.60) were the same as for the first fit (*e* = 0.59), discarding the possibility that the higher values of the error found for *a* > 0 could be non significant considering the uncertainties limits of the initial mode of telomere length distribution experimentally measured (260 ± 16 bp) and used as an input parameter.

We also estimated the lower bound of the error *emin* and the typical error *etyp* between one simulation and all the others. The variance of *N* = 1000 simulations immediately yielded *emin* = 0.22 ± 0.005, which represents the intrinsic variability of the simulated data. However, *emin* does not provide an estimate of the expected error between a specific set of data (whether experimental or simulated) and the bulk simulations. We thus calculated the error of one simulation compared to the others, which is formally the same calculation as for *e* but with simulated data instead of experimental ones, and we found *etyp* = 0.35 ± 0.10. We notice that the errors of the fits with our model are significantly greater than *etyp* suggesting that there may be some mechanisms that we have not taken into account in the model. Nonetheless, because the experimental data was completely enclosed within the envelope of our simulations (Fig. 2c), the model still captured the major mechanisms driving replicative senescence.

To take the precision of 3′-end overhang measurement into account^41^, we also tested a shorter overhang length (5–7 nucleotides) in the model and found *a* = 0, *Lmin* = 47 and *e* = 0.45 (Fig. S4), suggesting that a lower shortening rate of 3 bp/division might be slightly more consistent with the experimental data. However, even here, no contribution of the second shortest telomere could be found.

Taking all these fits together, the values of *a* and *e* were robust, in contrast to *Lmin*, which fluctuated depending on the input parameters. Thus, our model robustly reproduced the experimental timings of senescence onset, notably its heterogeneity, and reinforced the idea that only the shortest telomere contributed to signal senescence.

### Average telomere shortening in a population of senescent cells

**Figure 3:**
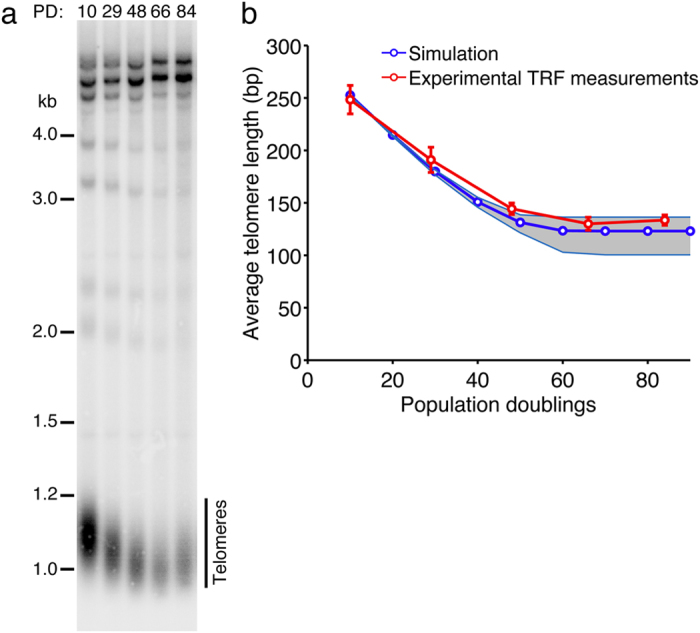
Telomere shortening dynamics at the population level. (**a**) Representative TRF Southern blot showing telomere shortening after telomerase inactivation. *Xho* I-digested terminal restriction fragments (TRF) were probed with a radioactive telomeric probe and average telomere length was measured. (**b**) Comparison between experimental measurements of the average telomere length as in (^a^) (*n* = 3 independent experiments, the error bars represent the standard deviation) with simulation of the dynamics of the average telomere length in a population of telomerase-negative cells assuming *Lmin* = 19 bp. The upper and lower limits of the simulation defining the uncertainty region (grey-shaded area) were derived from the cases *Lmin* = 35 and 2 bp, respectively.

In a population of senescent yeast cells, the average telomere length would reach over time a plateau at 80–140 bp^18^ (Fig. 3a). Given this average value for all telomeres, a threshold for the shortest telomere at *Lmin* = 19 bp (or at least between 2 and 35 bp) may not be intuitive because it is not trivial to derive the length of the shortest telomere from the large spread of telomere length distribution. To challenge our model, we predicted the shortening dynamics of the average of all telomeres by simulations with our model using a single threshold constraint on the shortest telomere and compared it to experimental measurements.

To do so, we simulated the divisions of a population of cells using a simple branching model, starting with a mean telomere length of 260 bp. As type A cells display homogeneous and constant cell cycle durations until senescence onset (Fig. 2a)^40^, we chose a binary behavior for simulated cells, being either in a dividing or an arrested state. A cell arrests when its shortest telomere reaches the *Lmin* = 19 bp threshold. At different time points, the length of all the telomeres in the population was averaged and recorded. This procedure mimicked the experimental assay in which telomere length was measured by TRF Southern blot. The simulated average telomere length decreased linearly over the first ~40 population doublings with a rate of ~3.5 bp/generation and then stabilized to reach a plateau at ~130 bp (Fig. 3b). This result closely matched the experimental telomere length dynamics using a telomerase-inactivated strain and TRF analysis (Fig. 3b). The threshold value of *Lmin* = 19 bp used for the shortest telomere in this simulation was thus consistent with and validated by biological data.

Strikingly, in the simulation, the threshold rule for the shortest telomere alone could constrain the dynamics of the average telomere length of the whole population of cells. Indeed, the average telomere length reached a plateau because of a competition effect: cells that reached senescence later were constantly selected over time and would have an average telomere length in the range of the plateau value. This competition effect reflects the complex dynamics of telomere shortening leading to a heterogeneous mixture of cells as they progress toward senescence.

### Interclonal variations partially contribute to senescence heterogeneity

We formulate two hypotheses to explain the experimental heterogeneity. First, heterogeneity in senescence timing may be explained by interclonal variations of the shortest telomere length in the founder cells, in which telomerase was inactivated. The other hypothesis involves an intrinsic variability of telomere length generated over the course of senescence. To discriminate between these two possibilities, we reasoned that the heterogeneity due to cell-to-cell (interclonal) variations would be suppressed if a single founder cell, instead of different independent ones, were used to produce multiple senescent cell lineages. In contrast, the intrinsic variability of senescence onset would be the same in both cases.

**Figure 4:**
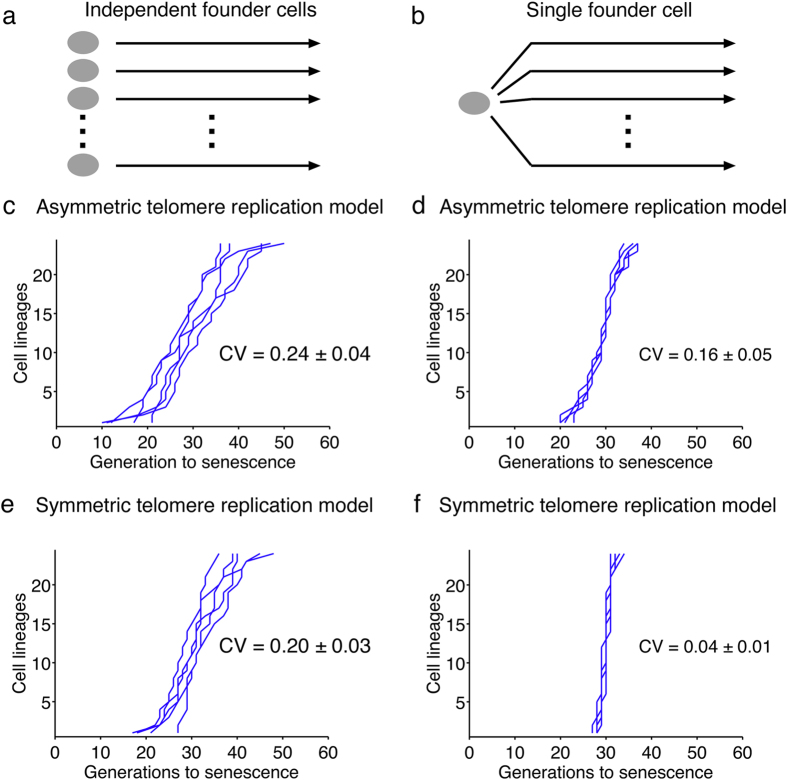
Contribution of interclonal variations and the asymmetry of telomere replication to senescence heterogeneity. (**a**) The scheme for the simulations used in (**c**,**e**) is pictured, showing independent founder cells for each lineage. (**b**) The scheme for the simulations used in (**d**,**f**) is shown and depicts a single founder cell from which multiple lineages are derived. (**c**) Representative set of 5 simulations starting from independent founder cells and using a model of asymmetric replication of telomeres (standard model). For (**c**–**f**), the mean and standard deviation of the coefficients of variations are indicated (CV, *n* = 55 independent simulations of 24 lineages). (**d**) Representative set of 5 simulations starting from a single founder cell and using a model of asymmetric replication of telomeres. (**e**) Representative set of 5 simulations starting from independent founder cells and using a model of symmetric replication of telomeres (modified model). (**f**) Representative set of 5 simulations starting from a single founder cell and using a model of symmetric replication of telomeres.

We thus simulated multiple cell lineages using the same initial cell, with its specific telomere length distribution, and analyzed the heterogeneity of the subsequent senescence onset profile by calculating the coefficient of variation (CV), defined as the standard deviation of the set of senescence onset timings divided by their mean (Fig. 4a,b). As expected, the simulation with a single founder cell did not fit the experimental data well because the actual experiment used different independent founder cells (Fig. 4d, compare with Fig. 4c). We calculated the coefficient of variation and found *CV* = 0.16 ± 0.05. In striking contrast, the simulations in Fig. 2, in which 24 independent founder cells were used, corresponded to a greater coefficient of variation of 0.24 ± 0.04 respectively (Fig. 4c). The decrease of CV upon using a single founder cell in the simulation showed that interclonal variations of telomere lengths contributed to the heterogeneity of senescence onset. However, we could still appreciate substantial variations in senescence onset in the clonal experiment simulations, suggesting the existence of yet another source of intraclonal noise (Fig. 4d), which seemed to be intrinsically captured in our model.

### The asymmetry of telomere replication generates stochasticity in telomere shortening dynamics

The only mechanism implemented in our model that might lead to differences in daughter cells regarding senescence onset would be the telomere strand asymmetry due to the 3′-end single-stranded DNA overhang and the distinct machineries involved in the replication in the two strands, namely the leading and lagging strand replication machineries (Fig. 1a). Indeed, asymmetric replication of telomeres produces two new telomeres of different lengths (one equal to the parental telomere, the “lagging telomere”; the other shorter by 5–10 bp, the “leading telomere”;^41^) so that, at the level of an individual telomere and cell division, telomere shortening is not constant but probabilistic with an all or nothing pattern.

To investigate the contribution of asymmetric telomere replication to senescence heterogeneity, we slightly modified our model by forcing, for each telomere replication, the two newly replicated telomeres to have the same length, which is the parental length minus the average shortening rate of the standard model. We then tested this artificial symmetric telomere replication model starting with a single founder cell (Fig. 4f). We found virtually no variations in senescence onset for all the lineages (*CV* = 0.04 ± 0.01, compared to *CV* = 0.16 ± 0.05 in the case of asymmetric telomere replication), showing that we managed to remove nearly all sources of variations. The decrease in the coefficient of variation with this symmetric model could barely be observed when we simulated independent founder cells, as it was largely masked by the interclonal variations (*CV* = 0.20 ± 0.03, compared to *CV* = 0.24 ± 0.04 for the asymmetric model) (Fig. 4e). Thus, in our mathematical model, the asymmetry of telomere replication directly affects senescence heterogeneity by modulating telomere-shortening dynamics in distinct lineages, even starting with the same founder cell.

### Initial identity of the shortest telomere at senescence onset

Theoretically, by this asymmetric replication, the shortest telomere is maintained in one of the two daughter cells and shortened in the other. In consequence, in some rare lineages, the shortening rate of the shortest telomere may be much lower than average and in others, much higher, thus explaining the intrinsic variations. However, the picture may be more complex: the shortening dynamics of the other telomeres has to be taken into account. For example, in a specific lineage, if the lagging telomere of the initial shortest telomere is inherited for several divisions in a row, the second shortest telomere may reach at some point the same length as the shortest and eventually switch rank with it and become the “new” shortest (See examples in Supplementary Movie). Indeed, stating that the shortest telomere signals senescence does not necessarily imply that this specific telomere was *initially* the shortest telomere when, many divisions earlier, telomerase was inactivated.

**Figure 5:**
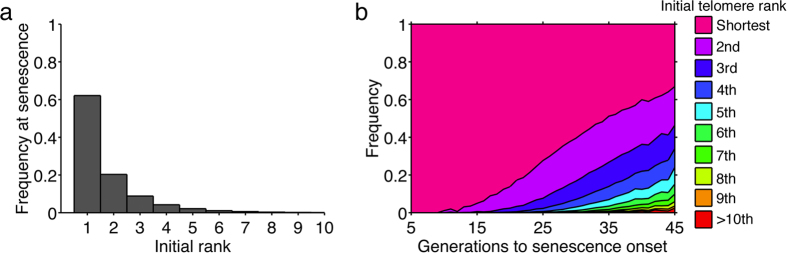
Telomere rank switching events involving the shortest telomere. (**a**) Aggregate frequency of rank switching events, i.e. frequency at which the initial n^th^ shortest telomere eventually becomes the shortest at senescence onset. Data derived from 10 000 simulations. (**b**) Frequency of rank switching events as a function of the generation of senescence onset. The initial telomere rank is indicated by its color.

To quantify the frequency of these “rank-switching” events, we used the simulation shown in Fig. 2 and traced back the origin of the signaling shortest telomere to find its initial rank in the founder cell, in which telomerase had been inactivated. Unexpectedly, in nearly 40% of the lineages, the initial shortest telomere was not the shortest at senescence (Fig. 5a). For instance, in ~20% of the cases, the initial second shortest telomere eventually became the shortest (Fig. 5a).

We then investigated the dependence of this telomere rank-switching phenomenon on the timing of senescence onset (Fig. 5b). We found that the short-lived lineages were essentially determined by their initial shortest telomere whereas for longer lineages, the initial second, third, etc., contributed increasingly more to signal arrest at the transition to senescence (Fig. 5b, Supplementary Movie). Nevertheless, the initial shortest telomere always maintained a major role in signaling senescence (Fig. 5a,b), consistently with previous work^20^. This simulation result suggested that *in vivo* short-lived and long-lived senescent lineages may experience different telomere shortening dynamics, again highlighting the complexity and heterogeneity of senescence dynamics.

## Discussion

Replicative senescence is a highly asynchronous and heterogeneous process. Characterizing the source of the underlying heterogeneity has important and broad implications that range from fundamental understanding of senescence signal and the escape from it in survivors or cancers to improvement of diagnosis and treatment of telomere-related diseases. Previous mathematical models of telomere shortening focused on proliferation potential and on factors that regulate telomere state and length, in populations of cells and mostly in mammalian cell cultures, in which complex pathways interact to control cell proliferation^22^,^29^,^43^,^44^,^45^,^46^,^47^,^48^,^49^,^50^,^51^,^52^. In this study, we break the overall senescence heterogeneity down into mechanistically distinct components in an arguably simpler model for telomere-induced replicative senescence, *S. cerevisiae*. To do so, we modeled the telomere shortening mechanism at the molecular level using the most accurate measurement of the 3′-end overhang, taking the recent experimental demonstration of the asymmetry of telomere replication into account^41^, and its consequences on senescence signaling at the level of individual cell lineages. Simulating and analyzing single-cell lineages instead of populations of cells were necessary to accurately assess senescence heterogeneity and we took advantage of single-lineage data of telomerase-negative yeast cells undergoing replicative senescence to fit and support our simulations^40^.

By comparing the simulations based on the model to the experimental data, we could test hypotheses on the nature of senescence signal. We found that there was robustly no contribution of the second shortest telomere to senescence, quantitatively reinforcing the idea that the shortest telomere reaching a threshold is sufficient to signal senescence^20^,^34^. We could also show by simulations that this rule constrains the division dynamics of senescent cells in a population, thus explaining the two-phase shortening pattern of the average telomere length dynamics^18^ (Fig. 3b).

After establishing the nature of senescence signal, we went on characterizing the different sources of heterogeneity. Clearly, cell-to-cell variations in the initial telomere distribution, due to the dynamics of shortening and lengthening by telomerase occurring independently at each telomere^32^, contribute to senescence heterogeneity but only partially. These interclonal variations were suppressed when we analyzed by simulations the progeny of a single founder cell instead of lineages from independent founder cells. Strikingly, in this condition, we still observed important variations in senescence onset timings and we could attribute them to the asymmetry of telomere replication mechanism. Indeed, this asymmetry is a molecular source of noise leading to probabilistic telomere shortening at the level of individual telomeres. Consequently, individual telomere shortening rate may be different in different cell lineages. Furthermore, not only does this mechanism modulate the shortening rate of the initial shortest telomere, but it also leads to telomere rank-switching events (Supplementary Movie). To illustrate how strongly this mechanism affects senescence dynamics, let us consider two opposite examples. In the specific cell lineage that systematically inherits the strand replicated by the leading-strand machinery of shortest telomere, thus shortening by ~5–10 bp at each division, replicative senescence may occur as fast as ~10 divisions. In contrast, in the long-lived lineages, the successive cells often inherit the G-strand of the shortest telomere, thus maintaining its length. But eventually, the second shortest (and afterwards even the third, fourth, etc.) reaches the length of the shortest and switches rank with it. Therefore, in the long-lived lineages, the two or three shortest telomeres tend to shorten at a slow rate, and the cell lineage can divide for more than 50 divisions until senescence. Overall, short-lived and long-lived lineages may experience very different telomere shortening dynamics and thus senescence dynamics, with potential consequences in the mechanisms of senescence arrest and in cell physiology.

In our related work^40^, some lineages displayed stochastic reversible cell-cycle arrest events, a behavior clearly distinct from type A lineages, which we termed type B. Because we could not reliably propose a characterized molecular mechanism to explain commitment into type B lineages, we decided to take only type A lineages into account for the present study. Type B lineages strongly contributed to experimental replicative senescence heterogeneity as they represent ~40% of the lineages and showed unpredictable cellular behaviors. However, at the scale of the population of senescent cells, type B lineages are negligible because of their lower fitness. We suggested that they undergo cycles of telomere damage and repair, which could stem from stochastic damage, external stress or mitochondrial dysfunction, globally increasing genome instability. In future works, it would be highly informative to model possible mechanisms of telomere dynamics in type B lineages to quantitatively study their contribution to senescence variability.

In conclusion, it is tempting to speculate that creating a diversity of senescence dynamics for different lineages may be a bet-hedging or a differentiation strategy. Conversely, the heterogeneity generated through telomere shortening may be crucial for genetic, epigenetic or metabolic changes, such as cancer transformation in mammals, which corresponds to rare events occurring to rare cells. Such considerations may not be relevant to wild-type yeast cells as they naturally express telomerase but because replicative senescence is inherently associated with important variability, wired in the structure of telomeres (asymmetry of telomere replication), evolution may have harnessed this variability in multicellular organisms for functional reasons.

## Materials and Methods

### Determination of the asymptotic law of telomere lengths when telomerase is active

The probability of action of telomerase depends on two parameters *β* and *L*_0_. For our purpose, we want to fit them from only two experimental steady state parameters of telomere length distribution: the mode and the skewness. From a mathematical point of view (*L*_*n*_) is a (time-homogeneous) Markov chain. As the cells have reached a steady state telomere length distribution before telomerase inactivation^40^, we assume that the telomere length distribution is the stationary distribution of this Markov chain. The probabilities of transitions as well as the equation defining the stationary distribution can be explicitly written. By a convergent iterative process, we are able to compute this stationary state. In particular, we can compute its mode and its skewness. By varying the two parameters *β* and *L*_0_, we determine the parameters giving the predefined mode and skewness.

### Determination of the weight *a* of *L*_2_ and of the threshold *Lmin*

Assume that the parameters *a* and *Lmin* are fixed. We pick 24 cells with telomere lengths according to the stationary distribution of the Markov chain (*L*_*n*_). We simulate the evolution of the 32 telomere lengths for each of the 24 subsequent lineages according to the model, and we record for each one the generation of senescence onset. This gives 24 increasingly ordered integers 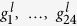. The integer number *l* refers to the number of the simulation. We repeat this simulation *N* = 1000 or *N* = 5000 times.

The error *e*(*a, Lmin*) between these numerically obtained generations of senescence onset and the experimental ones, ordered increasingly 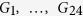, is measured by the root mean square deviation:
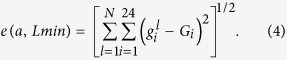


We repeat these simulations and calculations for different values of *a* and *Lmin*.

### Terminal restriction fragment (TRF) Southern blot

Genomic DNA was extracted from exponential growth cultures using a standard phenol:chloroform:isoamyl (25:24:1) alcohol purification procedure and ethanol precipitation. Then 1 μg of genomic DNA was digested with *Xho* I and the products were ethanol precipitated, resuspended in loading buffer (10 mM Tris pH 8.0, 1 mM EDTA, 5% glycerol, 0.04% xylene cyanol FF), and resolved on a 1.2% agarose gel for 14 h at 60 V. The gel was then soaked in a denaturation bath (0.4 M NaOH, 1 M NaCl) for 30 min and transferred by capillarity action to a charged nylon membrane (Hybond XL, GE Healthcare). The telomere-specific oligonucleotide probe (5′-GGGTGTGGGTGTGTGTGGTGGG-3′) was ^32^P-labeled at the 5′ terminus with ATP (γ-^32^P) and T4 polynucleotide kinase (New England Biolabs). The membrane was hybridized using the Rapid-hyb buffer protocol (GE Healthcare). In brief, the membrane was prehybridized at 42 °C in Rapid-hyb buffer for 1 h, then the radioactive probe (20 pmol) was added, and the incubation was continued for 1 h. The membrane was washed consecutively with 2 × SSC, 0.5% SDS (42 °C for 10 min); 2 × SSC, 0.1% SDS (42 °C for 20 min); and 0.1 × SSC, 0.1% SDS (20 °C for 30 min). The membrane was then imaged with a Typhoon FLA 9500 scanner (GE Healthcare).

## Additional Information

**How to cite this article**: Bourgeron, T. *et al*. The asymmetry of telomere replication contributes to replicative senescence heterogeneity. *Sci. Rep.* **5**, 15326; doi: 10.1038/srep15326 (2015).

## Supplementary Material

Supplementary Information

Supplementary Movie
