# Supplementary material for: The asymmetry of telomere replication contributes to replicative senescence heterogeneity
Source: Sci Rep. 2015 Oct 15;5:15326. doi: 10.1038/srep15326 (PMC4606794; doi:10.1038/srep15326)
Supplement: Supplementary Information [file srep15326-s1.pdf]

Supplementary Data

Title: The asymmetry of telomere replication contributes to replicative senescence heterogeneity.

Thibault Bourgeron[a,1], Zhou Xu[b,1,*], Marie Doumic[a,2,*] and Maria Teresa Teixeira[b,2]

Affiliations:

[a]INRIA Paris-Rocquencourt, Domaine de Voluceau, BP 105, 78153 Le Chesnay, France. UPMC University of Paris 6, JL Lions Lab., 4 place Jussieu, 75005 Paris, France.

[b]Centre National de la Recherche Scientifique, UMR8226, Laboratoire de Biologie Moléculaire et Cellulaire des Eucaryotes, Institut de Biologie Physico-Chimique, 75005 Paris, France. Sorbonne Universités, UPMC Univ Paris 06, UMR8226, Laboratoire de Biologie Moléculaire et Cellulaire des Eucaryotes, Institut de Biologie Physico-Chimique, 75005 Paris, France.

Supplementary Figures and Figure Legends

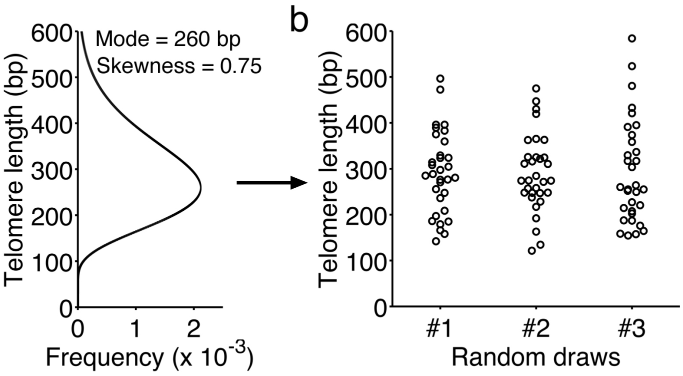

Figure S1. Initial telomere length distribution. (a) Simulation of the steady-state telomere length distribution corresponding to the strain TetO2-*TLC1* yT528, which had a mode of 260 ± 16 bp. (b) The distribution in (a) is used to randomly draw the lengths of 32 telomeres of the initial cell of a lineage, in which telomerase is inactivated. Three random draws are shown.

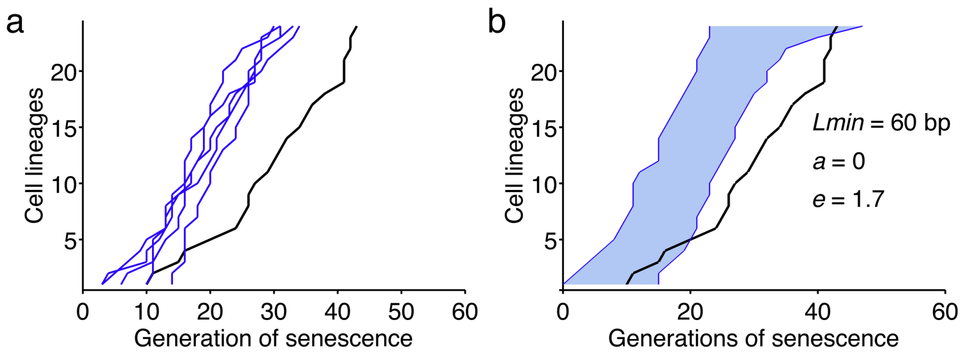

Figure S2. Example of a fit with an error of 1.7. (a) Representative set of 5 simulations of 26 independent lineages using $Lmin = 60$ bp and $a = 0$ compared to the experimental senescence onset timings (black line), which gives an error of $e = 1.7$. (b) Envelope of $n = 1000$ simulations (blue-shaded area) as in (a).

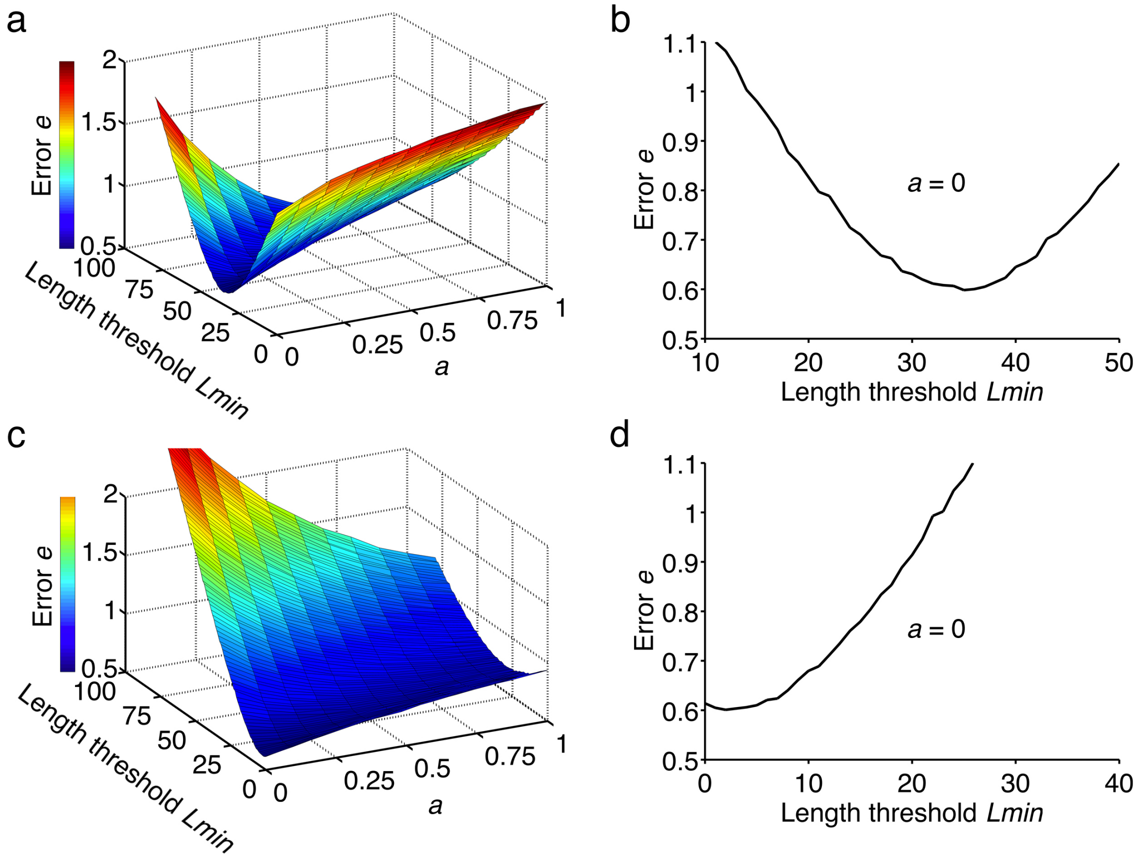

Figure S3. Fits using the upper and lower limits of the mode of the initial telomere length distribution. (a) 3D-plot of the error of the fit e as a function of a and $Lmin$, starting with an initial telomere length distribution of mode 276 bp. The minimum error is found for $a = 0$ and $Lmin = 35$ bp. (b) 2D-plot of the error of the fit e as a function of $Lmin$ for $a = 0$, starting with an initial telomere length distribution of mode 276 bp. (c) Same as (a), starting with an initial telomere length distribution of mode 244 bp. The minimum error is found for $a = 0$ and $Lmin = 2$ bp. (d) Same as (b), starting with an initial telomere length distribution of mode 244 bp.

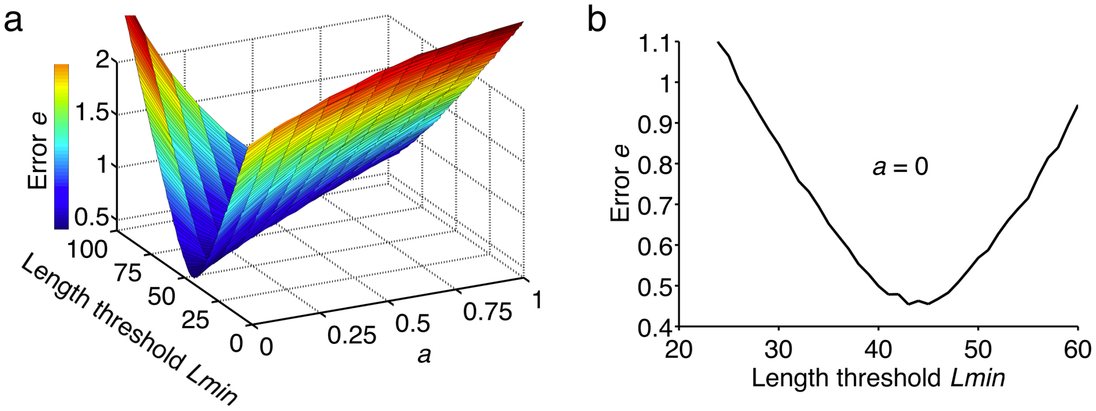

Figure S4. Fits using an overhang length with a uniform distribution on the set $\{5, 6, 7\}$, leading to a lower average shortening rate (3 bp). (a) 3D-plot of the error of the fit e as a function of a and $Lmin$. The minimum error is found for $a = 0$ and $Lmin = 47$ bp. (b) 2D-plot of the error of the fit e as a function of $Lmin$ for $a = 0$.

Supplementary Movie Legend

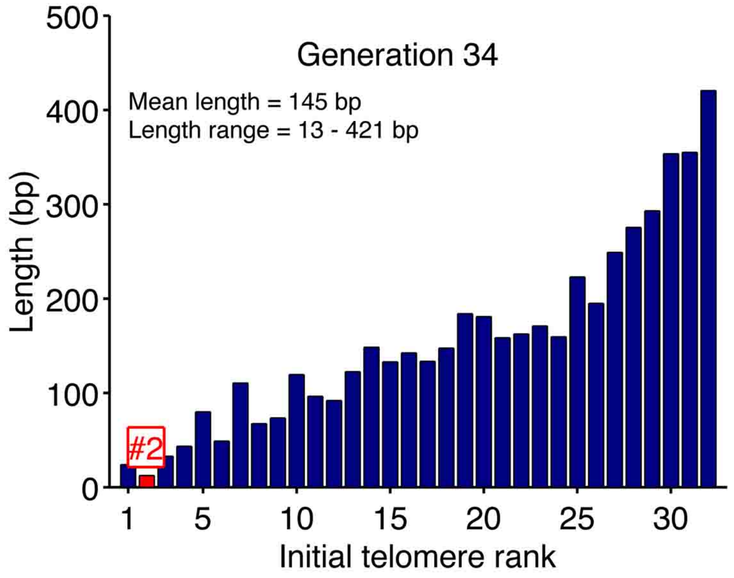

Snapshot of the movie

Shortening dynamics of 32 individual telomeres in independent lineages. A sample of the simulations performed for Fig. 2 is displayed as a dynamic movie showing the shortening of the 32 telomeres in a cell lineage ($n = 20$ independent lineages). Each part of the movie corresponds to a new lineage with a starting telomere set randomly drawn as in Fig. S1b and ordered on the x-axis. These 32 telomeres then evolve with cell divisions and the asymmetry of telomere replication combined to the random choice of one of the two daughter cells at each division (Fig. 1b) makes individual telomere shortening probabilistic. At any time, the shortest telomere is depicted is red. Thus, rank-switching events can be easily visualized. When the shortest telomere reaches the threshold $Lmin = 19$ bp, the simulation is stopped and the initial rank of the shortest telomere is indicated in red.
